# Cumulative Dopamine Genetic Score predicts behavioral and electrophysiological correlates of response inhibition via interactions with task demand

**DOI:** 10.3758/s13415-019-00752-w

**Published:** 2019-12-04

**Authors:** Sören Enge, Mareike Sach, Andreas Reif, Klaus-Peter Lesch, Robert Miller, Monika Fleischhauer

**Affiliations:** 1 Department of Psychology, Faculty of Natural Sciences, MSB Medical School Berlin, Calandrellistraße 1-9, 12247 Berlin, Germany; 2 Faculty of Psychology, Technische Universität Dresden, Dresden, Germany; 3 Department of Psychiatry, Psychosomatic Medicine and Psychotherapy, University Hospital Frankfurt, Frankfurt am Main, Germany; 4 Division of Molecular Psychiatry, Laboratory of Translational Neuroscience, Center of Mental Health, University of Würzburg, Würzburg, Germany; 5 Laboratory of Psychiatric Neurobiology, Institute of Molecular Medicine, I.M. Sechenov First Moscow State Medical University, Moscow, Russia; 6 Department of Translational Neuroscience, School of Mental Health and Neuroscience, Maastricht University, Maastricht, The Netherlands

**Keywords:** Genetic score, Dopamine, Response inhibition, ERP, NoGo-P3

## Abstract

Functional genetic polymorphisms in the brain dopamine (DA) system have been suggested to underlie individual differences in response inhibition, namely the suppression of a prepotent or inappropriate action. However, findings on associations between single DA polymorphisms and inhibitory control often are mixed, partly due to their small effect sizes. In the present study, a cumulative genetic score (CGS) was used: alleles previously associated with both impulsive behavior and lower baseline DA level, precisely the *DRD4* Exon III 7-repeat, *DAT1* VNTR 10-repeat and the *COMT* 158val allele, each added a point to the DA-CGS. Participants (N = 128) completed a Go/No-Go task varying in difficulty and EEG recordings were made with focus on the NoGo-P3, an ERP that reflects inhibitory response processes. We found a higher DA-CGS (lower basal/tonic DA level) to be associated with better performance (lower %FA and more adaptive responding) in the very demanding/rapid than in the less demanding/rapid condition, whereas the reverse pattern was true for individuals with a lower DA-CGS. A similar interaction pattern of DA-CGS and task condition was found for NoGo-P3 amplitude. In line with assumptions of distinct optimum DA levels for different cognitive demands, a DA-CGS-dependent variation of tonic DA levels could have modulated the balance between cognitive stability and flexibility, thereby affecting the optimal DA level required for the specific task condition. Moreover, a task demand-dependent phasic DA release might have added to the DA-CGS-related basal/tonic DA levels, thereby additionally affecting the balance between flexibility and stability, in turn influencing performance and NoGo-P3.

**Electronic supplementary material:**

The online version of this article (10.3758/s13415-019-00752-w) contains supplementary material, which is available to authorized users.

## Introduction

Inhibition or inhibitory control denotes the ability to effectively suppress stimuli, behavioral responses or impulses, habits, and memories that are currently irrelevant, interfering, incorrect, or inappropriate to perform goal-directed behavior. In a narrower sense, inhibition is considered being a part of executive functioning (Miyake et al., 2000), commonly distinguished in cognitive and motor forms of inhibition (Bari & Robbins, 2013). Cognitive inhibition refers to the inhibition of mental processes, such as memories, thoughts, or task-irrelevant stimuli, although the literature is inconclusive whether active inhibition does indeed take place on such cognitive levels or may at least partly be explained by other mechanisms such as selective attention (MacLeod, Dodd, Sheard, Wilson, & Bibi, 2003). On the other hand, it is widely agreed that inhibition exists on a motor level (Aron, 2007; Bari & Robbins, 2013). Specifically, response inhibition, namely the suppression of prepotent or inappropriate actions is considered a primary form of motor inhibition that shares an overlapping neural basis with other (cognitive) forms of inhibition and has been associated with various aspects of impulsivity-related behavior (Chamberlain, Fineberg, Blackwell, Robbins, & Sahakian, 2006; Cohen & Lieberman, 2010; Smith, Jamadar, Provost, & Michie, 2013). Generally, failure of inhibitory control has been repeatedly associated with pathologies, such as ADHD (Fisher, Aharon-Peretz, & Pratt, 2011; Nigg, 2001), substance abuse (Smith, Mattick, Jamadar, & Iredale, 2014), or obsessive-compulsive disorder (Chamberlain et al., 2006).

Concerning the neuromodulatory actions of inhibitory control, there is considerable evidence for dopaminergic (DA) signaling to play an important role (Buckholtz et al., 2010; Chambers, Garavan, & Bellgrove, 2009). Not surprisingly, therefore, studies also have tried to link genetic variations providing interindividual differences in central DA function to inhibitory control and impulsivity phenotypes (Benjamin et al., 1996; Gizer, Ficks, & Waldman, 2009; Li, Sham, Owen, & He, 2006; Nemoda, Szekely, & Sasvari-Szekely, 2011). In this vein, previous studies frequently focused on three functional polymorphisms: *DRD4* Exon III VNTR, *DAT1* VNTR, and *COMT* val158met (Chambers et al., 2009; Congdon & Canli, 2008; Gizer et al., 2009). However, findings on associations of single DA polymorphisms and inhibitory control are often mixed partly due to their small effect sizes and their insufficient statistical power when investigating multiple genetic variants simultaneously. To circumvent these issues, in the present study a cumulative genetic score of these genetic variants was examined with regard to behavioral and electrophysiological indicators of response inhibition and viewed in the light of recent models of DA function. The respective polymorphisms and the studies’ research agenda will be characterized as follows.

### Polymorphisms in the dopaminergic gene network and inhibition

Located in Exon III of the human dopamine receptor D4 (*DRD4*) gene, a 48 base pair (bp) variable number of tandem repeat (VNTR) functional polymorphism has been identified, with 2-repeats (2R), 4R and 7R being most common (Ding et al., 2002; Oak, Oldenhof, & Van Tol, 2000). Generally, the 7R allele has been associated with reduced receptor binding properties, usually compared to 4R or noncarriers of the 7R allele. DA levels had to be threefold higher to reach a similar level of D4 receptor functioning as the 4R protein (Asghari et al., 1995). The association of the 7R with less responsive D4 receptors is further supported by in vivo pharmacological studies (Froehlich et al., 2011; Hamarman, Fossella, Ulger, Brimacombe, & Dermody, 2004). Consequently, brain regions (primarily prefrontal regions) relying on the 7R receptor would require higher DA levels, which is assumed to contribute to impulsive phenotypes, such as risk-/novelty seeking or punishment behavior (Enge, Mothes, Fleischhauer, Reif, & Strobel, 2017; Swanson et al., 2000; Wang et al., 2004).

The gene coding for the DA transporter (DAT1, gene: *DAT1/SLC6A3*), expressed in prefrontal and striatal regions, contains a 40bp VNTR polymorphism in the 3'-untranslated region, with the most common alleles being 9R and 10R (Mitchell et al., 2000). A higher expression rate has been associated with the 10R allele. Higher DAT density results in less extracellular DA and thus lower DA levels, likely associated with the 10R allele (Heinz et al., 2000; Mill, Asherson, Browes, D'Souza, & Craig, 2002). However, inconsistent effects have also been reported (Costa, Riedel, Müller, Möller, & Ettinger, 2011; Van Dyck et al., 2005).

Lastly, the gene coding catechol-O-methyltransferase (COMT), an enzyme that plays a crucial role in prefrontal DA degradation (Käenmäki et al., 2010), contains a single nucleotide polymorphism (rs4680/val158met). The val allele of *COMT* val158met leads to an increase in COMT activity of approximately 35-50% and thus to a substantial decrease in dopaminergic activity and a lower DA tone compared with the met allele (Chen et al., 2004; Farrell, Tunbridge, Braeutigam, & Harrison, 2012; Tunbridge, Bannerman, Sharp, & Harrison, 2004).

At a behavioral and neurocognitive level, *DRD4* VNTR 7R, *DAT1* VNTR 10R, and *COMT* val158met val alleles have been associated with relatively lower inhibition function in inhibitory control tasks, as indicated by behavioral performance (Congdon, Lesch, & Canli, 2008; Cornish et al., 2005; Gizer & Waldman, 2012; Loo et al., 2003) and/or neural activation patterns (Congdon, Constable, Lesch, & Canli, 2009; Heinzel et al., 2013). Furthermore, all these alleles have been repeatedly linked to ADHD (Faraone, Doyle, Mick, & Biederman, 2001; Gizer et al., 2009; Kereszturi et al., 2008).

### Cumulative Genetic Scores (CGS)

Although these allelic variations in DA genes have been linked to inhibition and impulsive behavior, as with many studies on single polymorphisms, inconsistent and contradicting findings exist (Dresler et al., 2010; Gizer et al., 2009). Main reasons are the generally small effect sizes of single polymorphisms, making genetic effects difficult to detect (Nemoda et al., 2011; Witte & Flöel, 2012). A more recent approach trying to address such problems are so called *cumulative genetic scores* (CGS), also referred to as *multilocus genetic profile scores* or *polygenic scores* that have already proven successful in previous studies (Disner, McGeary, Wells, Ellis, & Beevers, 2014; Nikolova, Ferrell, Manuck, & Hariri, 2011; Pearson, McGeary, & Beevers, 2014): alleles associated with similar functional and/or behavioral effects are summed up to create a score representing a person’s accumulated dose of that specific (endo)phenotype. Because their effect is aggregated into a single score, the usually small effect sizes of polymorphisms are increased and therefore may be detected more readily, while avoiding type I error inflation due to multiple testing as well as unstable statistical models with too many genetic variables.

In the present study, each allele of the DA system associated with impulsive behavior added a point to the DA-CGS (*DRD4* VNTR 7R, *DAT1* VNTR 10R, *COMT* 158val), with a higher score representing an increased dose of impulsive behavior or reduced inhibitory control, respectively. Additionally, the DA-CGS may provide insights into the relationship between inhibition and DA on a functional system level, as a higher relative to a lower score presumably reflects lower brain-related baseline (tonic) DA levels, mainly in prefrontal regions, contributing to impulsive phenotypes, as outlined above. In line with the association of these alleles with both inhibitory deficits and putatively lower tonic DA levels, pharmacological studies in animals and humans demonstrated that impulsive behavior can be reduced by drugs that increase DA activity, especially in individuals with a high baseline impulsive behavior (De Wit, Enggasser, & Richards, 2002; Eagle, Tufft, Goodchild, & Robbins, 2007; Fernando et al., 2012). Therefore, low DA baseline (tonic) levels might be one factor underlying decreased inhibitory control. However, higher DA level seem not always to be beneficial in executive function tasks, suggesting a more complex relationship between DA and executive functioning (Cools & D'Esposito, 2011), which will be elaborated below.

### The Go/No-Go Paradigm and ERP indicators

A prototypical task frequently used to assess (motor) response inhibition is the Go/No-Go paradigm. In this task, individuals have to press a button in the majority of trials (Go trials) and have to refrain from doing so when a certain different stimulus (No-Go trials) appears. The frequently occurring Go trials establish a prepotent response tendency, which has to be inhibited during the randomly presented, low frequent No-Go trials. Thus, a button press on a No-Go trial suggests a failure in inhibitory control (i.e., false alarm; FA). To examine the rapid brain responses underlying response inhibition, the EEG has proven to be useful, providing a high temporal resolution. Seminal research by Falkenstein et al. (1999) and Bokura et al. (2001) revealed two event-related potentials (ERP) commonly elicited in the Go/No-Go task, which are hypothesized to reflect partly distinguishable inhibitory processes: The NoGo-N2 and the NoGo-P3, which will be addressed in the present study.

The NoGo-N2 is a negative ERP with a fronto-central scalp distribution peaking at around 200–400 ms after stimuli presentation that is reliably found to be larger in No-Go compared with Go trials (Bokura et al., 2001; Donkers & van Boxtel, 2004; Falkenstein et al., 1999; Jodo & Kayama, 1992). Furthermore, the NoGo-N2 has been shown to have a more negative-going peak and shorter latency in subjects with low rather than high FA rates and therefore has been thought of as an indicator of inhibitory control (Falkenstein et al., 1999). Nonetheless, the role of the NoGo-N2 is not undisputed, with other studies arguing it might rather reflect conflict processing (Donkers & van Boxtel, 2004; Enriquez-Geppert, Konrad, Pantev, & Huster, 2010). Summarizing the case for and against inhibition reflected in the NoGo-N2, Falkenstein (2006) concluded that, if this ERP reflects inhibition, it is rather premotor inhibition than motor response inhibition per se.

The NoGo-P3, a positive-going potential at 300–500 ms displays an anterior shift (central maximum) compared to the usually more parietal P3 (Bokura et al., 2001). Like the NoGo-N2, the NoGo-P3 is more pronounced in No-Go compared to Go trials, and a larger deflection usually coincides with more adaptive responding and lower FA rates, indicating more successful inhibitory processing (Falkenstein et al., 1999; Fisher et al., 2011). Other than the NoGo-N2, research increasingly suggests the NoGo-P3 to more directly reflect inhibition of a motor response (Enriquez-Geppert et al., 2010; Janette L. Smith et al., 2013).

### Research Questions

In our study, we addressed functional polymorphisms in the DA system with regard to individual differences in response inhibition. Previous studies of inhibitory control and impulsive behavior frequently focused on three functional DA polymorphisms: *DRD4* Exon III VNTR, *DAT1* VNTR, and *COMT* val158met (Congdon & Canli, 2008; Gizer et al., 2009). However, findings on associations of single DA polymorphisms and inhibitory control are often mixed due to their small effect sizes and their insufficient statistical power when investigating multiple genetic variants concurrently. We therefore applied a cumulative genetic score of these genetic variations, which we then examined with regard to behavioral and electrophysiological indicators of response inhibition. In the used Go/No-Go task, we varied task demands by limiting the time window for giving valid responses, providing a very demanding and a comparatively less demanding task to further examine the role of DA-CGS on inhibition performance under differing demands.

First, we expected to replicate findings of worse performance (i.e., higher error rates) and larger N2 and P3 deflections in No-Go compared with Go trials. However, because the NoGo-P3 is suggested to be more indicative of motor response inhibition, this study focused on NoGo-P3 amplitude, while the analysis of the relationship between DA-CGS and NoGo-N2 was exploratory in nature.

Second, drawing from the allele-specific effects of the single polymorphisms underlying DA-CGS calculation, as outlined above, one may expect a higher DA-CGS relative to a lower DA-CGS to be associated with lower inhibitory control as indicated by reduced inhibition performance and smaller NoGo-P3 amplitudes. As outlined above, however, the relation of DA tone with cognitive control seems to be more complex, as a large body of evidence suggests the existence of an optimum level of DA for cognitive control. That is, excessive or insufficient levels of DA may impair performance in cognitive control tasks, which is especially also depending on baseline (tonic) DA levels, as examined by the DA-CGS in the present study (Cools, Barker, Sahakian, & Robbins, 2001; Cools & D'Esposito, 2011). This assumption would point to role of interaction effects of varying DA level with task demands. Interaction effects might also be assumed because higher task difficulty may potentially increase (phasic) DA levels (Aalto, Brück, Laine, Någren, & Rinne, 2005; Benikos, Johnstone, & Roodenrys, 2013; Westbrook & Braver, 2016). That is, individuals with a lower DA tone (higher DA-CGS) might profit more from a difficult than from a relatively easier task condition compared to those with an already higher DA tone (lower DA-CGS). Within the framework of optimal DA levels for cognitive control, a prominent additional assumption is that distinct optimal DA levels exist, depending on the type of a cognitive task or task condition and the role DA plays in frontostriatal brain regions, thereby modulating the dynamic balance between cognitive stability and flexibility, required for adaptive cognitive control (for review see Cools & D'Esposito, 2011). Because the demanding Go/No-Go task condition requires rapid responding within a very short time frame, task performance could rely to a larger degree on a frequent and flexible updating and switching of attention between task-relevant representations. An increased flexibility might potentially ease the extremely rapid decisions required on stimuli in this condition, while a relatively strong cognitive stability would somewhat lower the responsiveness and high flexibility needed. Within the dynamic balance between flexibility and stability, however, a larger stability resulting in a stronger focus and maintenance of task-relevant representation could be overall more beneficial in the less demanding/rapid task condition relative to the demanding one, as an extremely rapid updating and responding is comparatively less required.

Since a higher DA tone (i.e., a lower DA-CGS) in the PFC is expected to increase stability, but decreases flexibility, and vice versa, DA-CGS-related differences in tonic DA levels (presumably in the PFC) could moderate the balance between stability and flexibility within frontostriatal circuits of cognitive control thereby contributing to possible condition-dependent differences (Bilder, Volavka, Lachman, & Grace, 2004; Cools & D'Esposito, 2011). A range of previous studies already provide evidence for these gene variations used to form the DA-CGS to potentially modulate the balance of stability and flexibility in cognitive control (Bilder et al., 2004; Gizer & Waldman, 2012). However, both, task-demand dependent phasic DA responses that add to tonic DA levels as well as the assumption that DA affects the balance between cognitive flexibility and stability can be integrated in the common framework of optimal levels of DA for different cognitive demands (Cooles and Esposito, 2011). Changes in phasic DA responses due to task demand could add to DA-CGS-dependent tonic DA levels, which in turn may additionally influence the balance between stability and flexibility and thus the optimal DA level for the task condition at hand. In sum, in the present study, especially interaction effects of the DA-CGS on inhibition behavior and NoGo-P3 amplitude are expected depending on the condition-specific variation of task demands. The above outlined assumptions of optimal DA levels for different cognitive requirements could serve to interpret such potential interactions of DA-CGS and task demand.

## Methods

### Sample

A total of 133 right-handed participants between the age of 18 and 35 (*M* age ± *SD*: 22.5 ± 3.6, 70 males) were recruited through advertisement on campus and during lectures from the student population of the Technische Universität Dresden. All participants gave written, informed consent for participation, and they were given course credit as compensation. All individuals were of central European origin and reported German as their mother tongue. No participant reported relevant health problems or underwent psychiatric or neurological treatment. All had a normal or corrected-to-normal vision and confirmed that they did not abuse drugs. Participants’ sleep duration as well as caffeine and alcohol consumption in the past 24 hours was assessed via self-report. Handedness was measured using the Edinburgh Handedness Inventory (Oldfield, 1971). Two participants were excluded from the final analysis based on their inferior behavioral performance, which will be explained in detail below. Another three had to be excluded from analysis due to missing or rare genotype data, resulting in 128 individuals that were included in the analyses (*M* age ± *SD*: 22.5 ± 3.6, 68 males). The procedure used in this study was in accordance with the principles of the Declaration of Helsinki (revised version) and was formally approved by the ethics committee of the Technische Universität Dresden.

### Procedure and the Go/No-Go Task

After being informed about the study, individuals received several questionnaires assessing sociodemographics, mood, and personality. Subsequently, participants were seated in an acoustically and electromagnetically shielded EEG cabin in front of a computer screen and the electrode cap was attached. EEG recording started with a 4-minute resting period with eyes open and closed (2-min each). Participants then completed the Go/No-Go task and afterwards three other short computerized tasks, which were part of another study and will be reported elsewhere. At the beginning of the Go/No-Go task, participants were instructed to respond as fast and accurately as possible by pressing the spacebar on a keyboard when any letter except the letter “X” appeared on the screen (Go trial) and to refrain from pressing any button when an “X” appeared (No-Go trial). Furthermore, participants were informed that the duration of presentation for each letter would vary throughout two different task blocks (conditions) and thus differed in their upper time limit for giving responses, providing a variation of difficulty. Afterwards, they completed a practice block of 25 trials and had the opportunity to ask questions. Specifically, the time period for giving valid responses was varied for two reasons: Pretests and previous findings showed that performance in inhibitory control tasks is modulated by age (Enge et al., 2014; Sweeney, 2001). Thus, regarding the present young student sample, the integrity of inhibitory control functions is expected to be at its peak. So, we aimed to create an overall challenging task to account for the on average high performance and consequently generate a sufficient FA rate, the primary measure of response inhibition in Go/No-Go tasks. Furthermore, we were interested in DA-CGS-dependent differences on varying task demands to account for theoretical and empirical knowledge on task type-dependent optimal DA levels in cognitive control. Therefore we designed and pretested a very demanding (400 ms) and a relatively less but still demanding condition (500 ms). Notably, time pressure is an essential factor to create a strong prepotent response tendency and has often been successfully applied in Go/No-Go paradigms (Jodo & Kayama, 1992; Janette L. Smith, Johnstone, & Barry, 2007). Each block consisted of 125 trials (20% No-Go) in which black letters (font: Arial, size: 72), subtending a visual angle of 0.76° horizontally and 1,05° vertically at a viewing distance of 60 cm, were presented in a pseudorandomized order at the center of a light gray screen. The inter-stimulus-interval was 1,000 ms. The task blocks were separated by a short break and presented in varying order that was counterbalanced across participants. Initial behavioral analyses revealed two participants with clearly deviant response patterns, suggesting that they did not follow or misunderstood the instructions for the Go/No-Go task. One was characterized by a close to 100% FA rate and the shortest mean RTs of all participants. The other displayed an extremely high rate of misses on Go trials (≥15%) and high FA rates coupled with noticeably prolonged RTs (in 400 ms blocks multiple standard deviations above average). Therefore, their data were excluded from further analysis.

### EEG recordings and pre-processing

Thirty-two Ag/AgCl electrodes, 29 of which affixed to an electrode cap (scalp sites: Fp1, Fp2, AF3, AF4, F7, F3, Fz, F4, F8, FC5, FC1, FC2, FC6, T7, C3, Cz, C4, T8, CP5, CP1, CP2, CP6, P7, P3, Pz, P4, P8, O1, O2), were used to continuously record EEG, vertical electrooculogram (VEOG), and horizontal electrooculogram (HEOG) at a sampling rate of 500 Hz. EEG recording was done using BrainVision Recorder software (Version 1.3 Brainproducts, GmbH, Munich, Germany). Two linked electrodes at left and right mastoids were used as reference and AFz as ground. A 0.1–250 Hz bandpass filter was applied and impedances were kept below 5 kΩ. Continuous EEG data was segmented into epochs from −200 ms to 1000 ms after stimulus onset. Epochs were then submitted to an infomax independent component analysis (ICA) using an infomax ICA function from EEGLAB (Delorme & Makeig, 2004) to remove artifacts from muscle and eye movements and electrical noise artifacts (Jung et al., 2000). Furthermore, the EEG data were lowpass filtered (30 Hz). Epochs were averaged separately for each participant, electrode, trial type (Go, No-Go) and task condition (400 ms, 500 ms). ERP components were measured relative to a 200 ms prestimulus baseline using BrainVision Analyzer software (Brain Products, Germany). N2 and P3 components were identified based on latency windows (N2: 160-350 ms; P3: 260–560 ms) and determined on the basis of grand averages and individual waveforms in each trial type.

### Genotyping and CGS calculation

DNA was extracted from saliva using the ORAgene DNA Extraction kit (DNA Genotek, Ottawa, ON, Canada) according to the manufacturer’s instruction and genotyped using routine PCR and RFLP following protocols described in detail elsewhere for *DRD4* Exon III, *DAT1* VNTR and *COMT* val158met (Congdon et al., 2009; Congdon et al., 2008)

In accordance with previous studies using CGS analysis, participants’ DA-CGS was formed by adding the numbers of alleles previously associated with impulsive behavior or reduced inhibitory control, respectively and functionally, with a lower DA tone, as outlined above, assuming a linear model; namely the number of *DRD4* VNTR 7R, *DAT1* VNTR 10R, and *COMT* (val158met) val alleles an individual carries. Consequently, the DA-CGS ranged from 0 to 6 (*M* ± SD: 2.77 ± 1.06), with the following frequencies: 0 (n = 1), 1 (n = 9), 2 (n = 46), 3 (n = 44), 4 (n = 19), 5 (n = 8), and 6 (n = 1).

To provide a descriptive comparison with the results of the DA-CGS, effects of the single polymorphisms also were examined. In accordance with previous studies, for *DRD4* Exon III VNTR, participants with at least one 7R allele (n = 38, i.e. 2/7 = 2, 3/7 = 1, 4/7 = 26, 6/7 = 1, 7/7 = 5, and 7/8 = 3) were contrasted against non-7R carriers (n = 90, i.e. 2/2 = 3, 2/4 = 13, 3/3 = 1, 3/4 = 11, 3/5 = 1, 4/4 = 59, and 4/6 = 2) (Dreber et al., 2009; Enge et al., 2017; Swanson et al., 2000). In terms of *DAT1* VNTR, based on previous research (Gurvich & Rossell, 2014), participants homozygous for the 10R allele (n = 77) were compared to the other *DAT1* VNTR genotypes (n = 51; i.e., 9/9 = 4, 9/10 = 44, and 10/11 = 3). The rare 6R allele (6/6: n = 1) was excluded from further analysis due to missing literature on its behavioral or functional relevance. In line with previous studies, individual analysis of COMT was conducted contrasting the three genotypes met/met (n = 38), val/met (n = 68), and val/val (n = 22) (Congdon et al., 2009; Strobel et al., 2011). Genotypes included in further analysis were in Hardy-Weinberg-Equilibrium (HWE): *DRD4* VNTR: χ^2^ = 25.40; df = 15; *p* = .092; *DAT1* VNTR: χ^2^ = 1.50, *df* = 3, *p* = .45; *COMT* val158met: χ^2^ = 0.71; *df* = 1, *p* = .48. HWE calculations were made using the HWxtest package (Engels, 2014) for R core.

### Statistical Analysis

Statistical analyses were performed using SPSS 22.0 for Windows (SPSS Inc., Chicago, IL). First, mean response times (RTs) for correct Go trials and false alarms (%FA) in No-Go as well as misses in Go trials were calculated for the 400 ms and 500 ms condition, respectively. The average percentage of misses was negligible (≤0.57%) and therefore not analyzed further.

To examine the relationship between DA-CGS and behavioral performance, the DA-CGS was submitted as covariate to two 2-way (block: 400 ms and 500 ms) repeated measures General Linear Models (GLM), one containing %FA and a second with mean RT as dependent variable. We further tested for confounding effects of sex, age, sleep duration, and caffeine as well as alcohol consumption during the past 24 hours for a significant influence on behavioral data. Only age (all others *p* > .05) correlated with RT (in the 400 ms condition, nonparametric *r*_*s*_ = .188, *p* = .032) and thus was subsequently considered as control variable in the respective GLM.

To investigate the electrophysiological effects, the DA-CGS was analyzed along with ERP measures. Because response inhibition exclusively takes place in No-Go trials, our analysis focused on ERPs of correct No-Go trials, which is in accordance with previous studies (Falkenstein et al., 1999; Janette L. Smith et al., 2007). We primarily concentrated on the NoGo-P3 as an electrophysiological correlate of response inhibition as proposed earlier (Enriquez-Geppert et al., 2010; Falkenstein et al., 1999). The NoGo-N2 was analyzed for exploratory reasons, as outlined above. Similarly, the latency of ERPs will be presented only for descriptive reasons. Aligning with previous findings, the NoGo-P3 displayed a shift towards more anterior electrodes and the NoGo-N2 was most pronounced at fronto-central sites (Bokura et al., 2001; Falkenstein et al., 1999). Consequently, in line with these studies, Cz and Fz were used for analysis of the NoGo-N2 and FC1 and FC2 for the NoGo-P3. The DA-CGS was submitted as covariate to the resulting 2 (condition: 400 ms and 500 ms) x 2 (electrode: Cz/Fz for N2; FC1/FC2 for P3) repeated measures GLMs. Age was considered as control variable and entered as covariate because significant and marginally significant correlations were observed with mean N2 (*r* = .19, *p* = .034) and P3 (*r* = −.15, *p* = .09) amplitudes.

Additionally, for exploratory comparison with the DA-CGS results, all GLMs were repeated with the single polymorphisms as predictors. Further, paired-sample *t*-tests were applied to compare N2 and P3 amplitude in No-Go vs. Go trials and Pearson correlations were calculated to investigate relationships between ERPs and behavioral measures.

## Results

### Behavioral data

As expected, the percentage of misses in Go trials was extremely low in both conditions (*M* ± *SD*: 400 ms: 0.59 ± 1.06%; 500 ms: 0.40 ± 1.13%). On average, the percentage of false alarms (%FA) was higher in the 400 ms (33.02 ± 15.07%) than in the 500 ms condition (29.21 ± 16.27%), indicating increased task demand in the 400 ms condition, as expected. The difference was significant, as assessed using a one sample *t*-test, *t*(127) = 2.737, *p* = .007. Similarly, mean RTs were lower in the 400 ms condition (328.32 ± 32.05) compared with the 500 ms one (337.48 ± 31.25), *t*(127) = −4.545, *p* < .001, due to a shorter time span for giving responses. Moreover, a speed-accuracy trade-off was observed; mean RTs were highly significantly negatively associated with %FA in both conditions, showing that participants who responded faster on Go trials made more mistakes on No-Go trials (400 ms: *r*_*s*_ = −.404, *p* < .001; 500 ms: *r*_*s*_ = −.386, *p* < .001, *N* = 128).

### ERPs

**Fig. 1:**
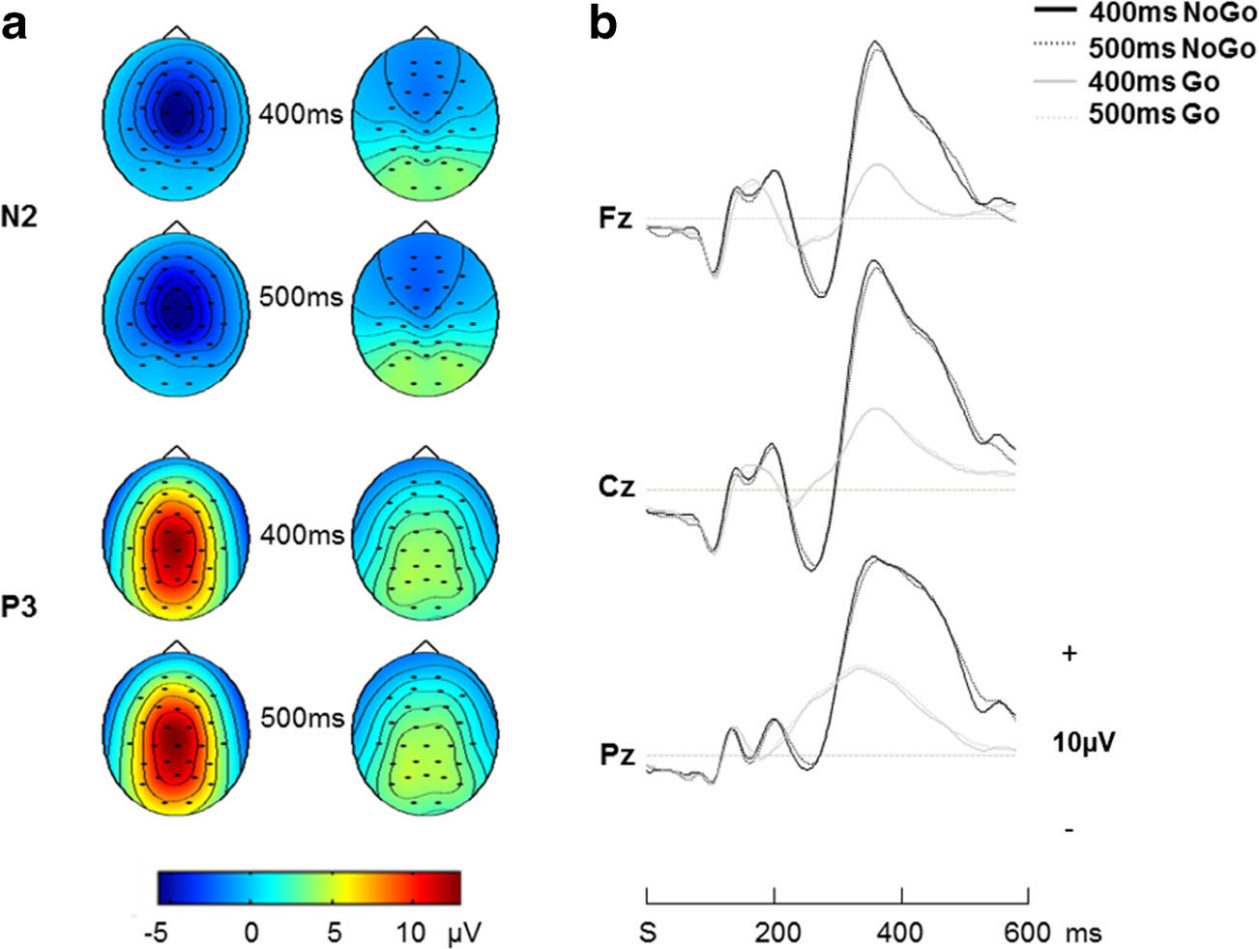
**A** Topographic Maps for N2 and P3 in No-Go and Go trials. **B** Stimulus locked grand mean waveforms for Go and No-Go trials for 400 ms and 500 ms separately (N = 128)

Previous findings of the NoGo-N2 and NoGo-P3 could be replicated. When compared using one-sample *t*-tests, N2 as well as P3 showed a greater peak amplitude in No-Go compared with Go trials (N2: Cz, Fz; P3: FC1, FC2) in each condition (all *p* < .001). An expected fronto-central distribution of the NoGo-N2 and the typical shift of the NoGo-P3 towards more anterior electrodes could be observed (Figure 1). Furthermore, the NoGo-N2 showed a longer latency compared to the N2 in Go trials in each of the two task conditions and for each of the two electrode positions (all *p* < .001). The same was true for the NoGo-P3 in the 500 ms condition, *t*(127)= −2.78, *p* = .006. The two No-Go ERPs were not correlated with each other (all *p* > .40, *N* = 128).

### ERPs and performance

In order to investigate the relationship between performance and electrophysiological measures, Spearman correlations were calculated for each ERP within the corresponding task conditions. Neither NoGo-N2 nor NoGo-P3 amplitude nor latency (averaged across the electrode positions) was significantly correlated with %FA (all *p* > .05, *N* = 128). However, RTs were associated with electrophysiological measures with correlations varying slightly across electrodes and trials. Shorter response times were associated with greater N2 amplitudes on correct NoGo trials (400 ms condition: *r* = .203; 500 ms: *r* = .293 averaged across Fz and Cz, all *p* < .05 with *N* = 128; for NoGo-N2 amplitude a positive correlation indicates a reversed relationship), as well as shorter latencies (400 ms condition: *r* = .470; 500 ms: *r* = .305, all *p* < .05, *N* = 128). The same was found for the NoGo-P3 peak (400 ms condition: *r* = −.284; 500 ms: *r* = −.247 averaged across FC1 and FC2, all *p* < .05, *N* = 128) and latency (400 ms condition: *r* = .410, 500 ms: *r* = .546, all *p* < .05, *N* = 128).

### DA-CGS and performance

**Fig. 2:**
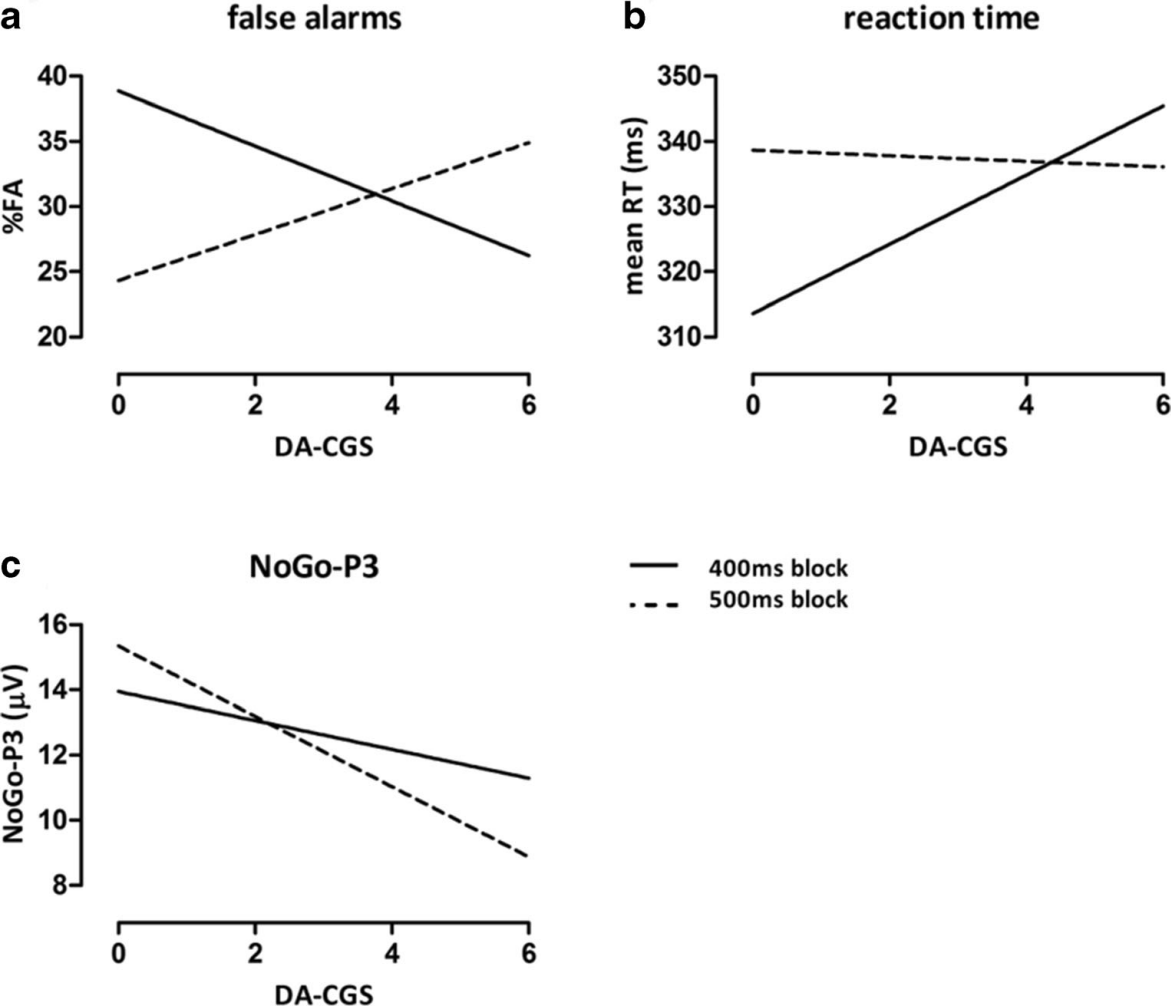
Influence of the DA-CGS on (**a**) percentage of false alarms (%FA), (**b**) mean response time on Go trials (RT) and (**c**) P3 amplitude on correct No-Go trials (NoGo-P3) in the different task blocks. For (**b**) and (**c**) age was included in the model

Next, we tested our main hypotheses regarding effects of DA-CGS on response inhibition. There was no main effect for the DA-CGS, neither for %FA, *F* (1,126) = 0.023, *p* = .881, *η*_*p*_^*2*^ < .001, nor mean RT, *F* (1,125) = 0.777, *p* = .380, *η*_*p*_^*2*^ = .006 (Table 1). However, a significant interaction between DA-CGS and task conditions occurred for %FA, *F* (1,126) = 9.24, *p* = .003, *η*_*p*_^*2*^ = .068. As depicted in Figure 2A, individuals with a higher DA-CGS showed a lower FA rate in the more demanding 400 ms block than in the 500 ms block, whereas the opposite was true for those with lower DA-CGSs. A similar interaction between the DA-CGS and task conditions was observed for mean RT, *F* (1,125) = 9.682, *p* = .002, *η*_*p*_^*2*^ = .072. A higher DA-CGS went along with prolonged RT in the more demanding 400 ms than in the 500 ms condition, while low DA-CGS individuals again showed the opposite pattern (Figure 2B). However, due to a much lower influence of DA-CGS on RT in the less demanding 500 ms condition, the disordinal interaction was less pronounced. For an additional depiction of the individual values, see Figures 1A and B in the supplementary material.

**Table 1:** Repeated measures ANOVA models of performance measures

	%FA			RT		
	*F*	*p*	*η*_*p*_^*2*^	*F*	*p*	*η*_*p*_^*2*^
*Main effects*						
Task condition	14.813	<.001	.105	3.323	.071	.026
DA-CGS	0.028	.881	<.001	0.777	.380	.006
*Interaction effect*						
Task condition × DA-CGS	**9.241**	**.003**	**.068**	**9.682**	**.002**	**.072**
*Effects of the control variable*						
Age				1.011	.317	.008
Task condition × Age				0.018	.894	<.001

DA-CGS was included as covariate in the model. Moreover, age was controlled for in the model of mean RT due to a significant association between age and RT.

%FA: percentage of false alarms; RT: response time on Go trials

We additionally analyzed the data using an extreme-group design where individuals with a DA-CGS of 0, 1, and 2 (n = 56) were compared to individuals with a DA-CGS of 4, 5, and 6 (n = 28) implementing the group variable as factor in the models. The interaction effects remained stable and even increased in effect size, for %FA, *F* (1,82) = 11.842, *p* = .001, *η*_*p*_^*2*^ = .126, as well as for RT, *F* (1,81) = 10.213, *p* = .002, *η*_*p*_^*2*^ = .112.

### DA-CGS and ERPs

In accordance with these behavioral findings and in line with expectations, an interaction effect of task condition and DA-CGS on NoGo-P3 amplitude was found, *F* (1,125) = 3.976, *p* = .048, *η*_*p*_^*2*^ = .031. A higher DA-CGS was associated with larger NoGo-P3 amplitudes in the more demanding condition than in the easier one, while the opposite was true for those with a lower DA-CGS (Figure 2C and Figure 1C in the supplementary material). Although the lines are crossing each other and thus the superiority of one condition over the other is not constant across the range of DA-CGS scores, the interaction was only semi-disordinal in nature as larger DA-CGS scores went along with lower No-Go P3 amplitudes in both conditions. The additional extreme-group comparison (see above) also revealed a significant interaction of DA-CGS × task condition on NoGo-P3 amplitude, *F* (1,81) = 5.584, *p* = .021, *η*_*p*_^*2*^ = .064, which was again larger in effect size than the effect gained by the DA-CGS entered as covariate. Neither NoGo-N2 amplitude nor NoGo-N2 or -P3 latency were associated with the DA-CGS (all *p* > .1, see Table 2 for P3 and Table 3 for N2).

**Table 2:** Repeated measures ANOVA models of NoGo-P3

	NoGo-P3 amplitude			NoGo-P3 latency		
	*F*	*p*	*η*_*p*_^*2*^	*F*	*p*	*η*_*p*_^*2*^
*Main effects*						
Task condition	0.089	.766	.001	0.001	.971	<.001
Electrode	0.510	.477	.004	1.283	.260	.010
DA-CGS	3.165	.078	.025	1.287	.259	.010
*Interaction effect*						
Task condition × DA-CGS	**3.976**	**.048**	**.031**	0.885	.349	.007
Electrode × DA-CGS	0.499	.481	.004	0.001	.982	<.001
*Effects of the control variable*						
Age	2.589	.110	.020	0.657	.419	.005
Task condition × Age	0.124	.725	.001	0.011	.916	<.001
Electrode × Age	0.255	.614	.002	1.338	.250	.011

DA-CGS was included as covariate in the model. Moreover, age was controlled for because of significant associations with ERP measures. Results refer to mean P3 amplitude and latency during correct No-Go trials. Due to readability, we refrained from depicting the Task condition × Electrode effects (all p > .05).

**Table 3:** Repeated measures ANOVA models of NoGo-N2

	NoGo-N2 amplitude			NoGo-N2 latency		
	*F*	*p*	*η*_*p*_^*2*^	*F*	*p*	*η*_*p*_^*2*^
*Main effects*						
Task condition	0.089	.766	.001	0.030	.864	<.001
Electrode	0.007	.935	<.001	0.279	.598	.002
DA-CGS	0.781	.378	.006	1.412	.237	.011
*Interaction effect*						
Task condition × DA-CGS	0.293	.589	.002	1.753	.188	.014
Electrode × DA-CGS	0.198	.657	.002	0.165	.686	.001
*Effects of the control variable*						
Age	1.188	.278	.010	0.473	.493	.004
Task condition × Age	0.207	.650	.002	0.045	.832	<.001
Electrode × Age	0.094	.760	.001	1.487	.225	.012

DA-CGS was included as covariate in the model. Moreover, age was controlled for because of significant associations with ERP measures. Results refer to mean N2 amplitude and latency during correct No-Go trials. Due to readability, we refrained from depicting the Task condition × Electrode effects (all p > .05).

### Descriptive comparison of individual DA polymorphisms with DA-CGS

For descriptive purposes only, the results of the repeated measures analyses containing *DRD4* Exon III, *DAT1* VNTR, and *COMT* val158met, respectively are given in Table 4. None showed a consistent relationship to behavioral and electrophysiological data comparable to the DA-CGS. Although when an interaction effect of a single polymorphism did reach significance, it pointed in the same direction as the respective CGS effect. Carriers of the 7R *DRD4* VNTR allele, which added a point to the CGS, displayed less %FA in the more difficult 400-ms than in the easier 500-ms block, whereas the opposite was true for non-7R carriers. Similarly, participants homozygous for the 10R allele of *DAT1* VNTR had a larger NoGo-P3 in the 400-ms than in the 500-ms block, whereas the effect was reversed for the other genotype carriers. Finally, individuals homozygous for the *COMT* 158val allele, which like the *DAT1* VNTR 10R allele contributed to a higher CGS, showed prolonged mean RTs in the 400 ms condition than in the 500 ms condition, whereas individuals with at least one met-allele showed the opposite pattern.

**Table 4:** Results from the repeated measures GLMs for the interactions of DA-CGS and single polymorphism with task condition

	%FA			Mean RT			NoGo-P3		
	*F* (*df*)	*p*	η_p_^2^	*F* (*df*)	*p*	η_p_^2^	*F* (*df*)	*p*	η_p_^2^
DA-CGS	**9.241 (1,126)**	**.003**	**.068**	**9.682 (1,125)**	**.002**	**.072**	**3.376 (1,125)**	**.048**	**.031**
DRD4 VNTR	**4.227 (1,126)**	**.042**	**.032**	1.314 (1,125)	.254	.010	0.036 (1,125)	.850	<.001
DAT1 VNTR	1.923 (1,126)	.168	.015	0.163 (1,125)	.687	.001	**4.023 (1,125)**	**.047**	**.031**
COMT Val158met	1.526 (1,125)	.222	.024	**9.416 (1,124)**	**<.001**	**.132**	2.545 (1,124)	.083	.039

## Discussion

In the present study, we investigated the relationship between genetic variations of DA function and response inhibition by behavioral and electrophysiological measures. Inconsistent results persist in studies focusing on single DA polymorphisms. Small effect sizes and insufficient statistical power to investigate multiple genetic variants simultaneously might underlie this heterogeneity (Gurvich & Rossell, 2014; Li et al., 2006; Nemoda et al., 2011; Witte & Flöel, 2012). To account for such limitations, we approached the topic using a CGS calculated by adding up the numbers of a person’s alleles that have been previously associated with both impulsive behavior and a lower DA tone at the DA system level (*DRD4* VNTR 7R, *DAT1* VNTR 10R, and *COMT* 158val). As this so called DA-CGS related more consistently to behavioral and EEG outcome measures than any single polymorphism our study supports the use of the CGS approach (Disner et al., 2014; Pearson et al., 2014). Additionally, as will be elaborated below, our results are in accordance with prominent assumptions of DA functioning.

### Behavioral performance and the DA-CGS

To assess response inhibition, participants completed a Go/No-Go task, with conditions varying in difficulty by the upper time limit for giving valid responses being either 400 ms (very demanding) or 500 ms (relatively less demanding). Consistently, participants committed significantly more %FA in the 400 ms condition compared to the 500 ms one. A lower FA rate was substantially correlated with slower RTs, suggesting the presence of a speed-accuracy trade-off. In other words, participants might have avoided erroneous responses on No-Go trials at the cost of slowing down on their responses on Go trials.

Further, as expected, the DA-CGS significantly interacted with task condition in predicting task performance. Individuals with higher DA-CGSs, presumably related to a lower DA tone and a higher proneness to impulsive behavior, showed lower FA rates as well as slower RTs in the more demanding 400 ms condition than in the 500 ms condition. As shown in Figure 2 (see also Figure 1 in the supplementary material), the opposite pattern was observed for individuals with lower DA-CGSs (i.e., a higher DA tone). They showed more adaptive responding (lower FA at higher RT or positive %FA difference values and negative RT difference values, respectively) in the less demanding 500 ms than in the 400 ms condition. So, when task demands were high, a presumably lower DA tone (high DA-CGS) was relatively more beneficial for response inhibition as indicated by more adaptive responding and lower FA rate, while relatively lower task demands reversed this relationship.

These results would potentially match previous findings demonstrating both beneficial and deteriorating effects on executive functioning after DA administration (for a review see Cools & D'Esposito, 2011). Wallace et al. (2011) for example found that the administration of a DA agonist aided performance in a working memory task for individuals with a low baseline working memory capacity, but impaired performance for those with an already high baseline capacity. This baseline working memory capacity is thought to partly reflect baseline DA levels, implying that individual differences in tonic DA levels could underlie mixed findings regarding the relationship between DA and behavior (Cools, Gibbs, Miyakawa, Jagust, & D'Esposito, 2008). As mentioned at the beginning, similar baseline behavior-dependent effects of DA drugs have been demonstrated for impulsive behavior (De Wit, Crean, & Richards, 2000; Eagle et al., 2007). In other words, whether an increase in DA signaling improves or impairs performance also depends on baseline (tonic) DA activity (Cools et al., 2001; Cools et al., 2009; Farrell et al., 2012).

In line with these findings, Cools and D’Esposito (2011) suggested an inverted u-shape relationship between DA and performance in cognitive control tasks. According to this view, an optimum level of DA for cognitive control exists, suggesting that insufficient or excessive DA level might impair performance on cognitive control. We also found a DA (baseline) dependent change in performance, moderated by the influence of the DA-CGS. However, we did not manipulate DA by drug administration. In the present study task demands were varied, which, however, might also affect DA transmission: It has been shown in some studies that demanding tasks might increase effort thereby increasing DA levels relative to a less demanding/rapid task (Benikos et al., 2013; Westbrook & Braver, 2016). Furthermore, higher task demands have been shown to increase activation in the substantia nigra – a major part of the DA brain system. This activation has been linked directly to an increase in DA release (Schott et al., 2008). On the same note, a PET study by Aalto et al. (2005) demonstrated increased DA release in a difficult two-back task relative to an easier zero-back task. Thus, in the present study increased task demands in the 400 ms condition may have potentially resulted in an increase in DA activity compared to the easier 500 ms one.

In accordance with the theory of an optimal level of DA for cognitive control (Cools & D'Esposito, 2011), a potential increase in (phasic) DA in the more difficult 400ms condition might have pushed the overall level of DA towards the optimum of functioning for individuals with a genetically driven lower tonic DA level (higher DA-CGS). In turn, this might have facilitated performance, indicated by fewer FAs. Conversely, for individuals with a higher tonic DA level (lower DA-CGS) the increase in DA may have led DA levels beyond the optimum and thus relatively impaired performance.

Generally, there is good evidence of different optimal DA levels in cognitive control, depending on type of cognitive task or task condition. These different DA levels required additionally influence the dynamic balance between cognitive stability and flexibility needed for adaptive cognitive control. Evidence from PET and fMRI studies in humans suggest that baseline-dependent effects of DA on cognitive control may mirror a DA-related modulation of frontostriatal connectivity (see Cools & D'Esposito, 2011). Because of a reciprocal dependency of DA transmission in these regions, an increase of DA in the PFC may promote a decrease of DA in the striatum, and vice versa (Akil et al., 2003; Durstewitz & Seamans, 2008; Meyer-Lindenberg et al., 2005). From a functional perspective, higher basal DA levels in the PFC are thought to promote cognitive stability, but impede flexibility, while the opposite is assumed for striatal DA. The tonic/phasic model of DA function previously suggested by Grace (1995) and Bilder et al. (2004) adds to this topic, because it assumes that increased tonic DA level associated with increased D1 and decreased D2 receptor activation could lead to a decrease of subcortical phasic DA responses. A lower DA tone on the other hand causes stronger subcortical phasic DA responses associated with opposite D1/D2 activation patterns.

Accordingly, genetic variations modulating tonic/basal DA function at the prefrontal cortical level have been shown to affect the balance between stability and flexibility in cognitive control. This has been repeatedly demonstrated for COMT val158met, with the met allele favoring performance in different executive function tasks that mainly require stability. Performance in task that require flexible updating, however, was compromised compared to val allele carriers, suggesting that high DA baseline levels in prefrontal areas may partially hamper cognitive flexibility in humans (Colzato, Waszak, Nieuwenhuis, Posthuma, & Hommel, 2010; M. J. Frank & Fossella, 2011; Krugel, Biele, Mohr, Li, & Heekeren, 2009; Nolan, Bilder, Lachman, & Volavka, 2004). Similar to COMT that is predominantly expressed in the PFC and that regulates the extrasynaptical degradation of DA, the D4 receptor mainly acts in frontal brain regions. Interestingly, the 4-repeat allele of DRD4 Exon III that contributes to a lower DA-CGS and exhibits increased D4 receptor sensitivity than the 7-repeat allele may possess comparable effects on stability/flexibility in humans than the met (vs. the val) allele of COMT (Gizer & Waldman, 2012; Logue & Gould, 2014; Muller et al., 2007). Consistently, pharmacological stimulation of D4 receptors in laboratory animals led to perseverative behavior, while D4 receptor antagonists improved cognitive flexibility. Thus, our DA-CGS effects on response inhibition could concur with such PFC-related DA effects on cognitive flexibility vs. stability in frontostriatal circuits (Cools & D'Esposito, 2011). This may also be supported by meta-analytic fMRI data demonstrating frontostriatal areas being typically recruited during Go/No-Go tasks (Criaud & Boulinguez, 2013; Simmonds, Pekar, & Mostofsky, 2008; Steele et al., 2013) and by the relationship between tasks of cognitive flexibility/stability and the inhibition function (Miyake & Friedman, 2012). Furthermore, conceptual work describes the imbalance between stability and flexibility as a control dilemma playing an overarching role for cognitive control including inhibitory control (Goschke, 2000; Hommel & Colzato, 2017).

Given that in the demanding 400 ms condition rapid responses are required within a very short time frame, task performance could rely more on a frequent and flexible updating of task-relevant information. That is, a heightened sensitivity and a flexible responding to task-relevant changes, respectively, would presumably be more beneficial to rapidly switch from Go-evoked prepotent responding in presence of rare NoGo events (Bilder et al., 2004; Nolan et al., 2004) in the 400 ms compared with the 500 ms condition. Hence, in the 400 ms condition, a higher DA tone (i.e., a lower DA-CGS) in the PFC might partly impede a flexible responding to task-relevant information, thus gradually lowering task performance relative to the 500 ms condition in these carriers. In the 500 ms condition, however, a higher DA tone in the PFC that promotes a higher cognitive stability and maintenance of task-relevant representations may be comparatively more beneficial for task performance as extremely rapid updating and responding are less required. Conversely, individuals with a higher DA-CGS (i.e., a lower DA tone), which may be associated with higher cognitive flexibility, show relatively better performance in the 400 ms than in the 500 ms condition.

In view of cognitive stability/flexibility and DA activity, there is converging evidence for both *COMT* val158met and *DRD4* Exon III that they modulate tonic DA particularly at the level of the PFC but rather not at the striatum. However, the role of the DAT1 VNTR alleles within the DA-CGS is less clear, as DAT is expressed both in the PFC and in the striatum, but is most abundant in the striatum. Whether and to what extent DAT1 VNTR alleles exert their effects on response inhibition either primarily in the PFC or in the striatum or, because of their interplay, in both can therefore not yet be conclusively clarified. The same issue has been discussed in other studies where additive effects of *COMT* val158met and *DAT1* VNTR during working memory tasks were only reflected in prefrontal cortical but not in striatal activation (Bertolino et al., 2006; Caldu et al., 2007). Nonetheless, on a behavioral level there are several studies as well as meta-analytic evidence that support associations of the 10R allele of *DAT1* VNTR (contributing to a higher DA-CGS) with impulsive phenotypes including response inhibition (Congdon et al., 2008; Gizer et al., 2009; Gizer & Waldman, 2012).

In sum, our results would potentially concur with recent findings demonstrating that higher task demand may cause a DA release that in turn may add to the DA-CGS-dependent tonic DA levels, which leads to the optimal level being reached or exceeded. This may be combined with accumulating evidence on the considered polymorphisms showing that the condition-specific effects on inhibition performance could reflect shifts in the balance of cognitive stability and flexibility. The DA-CGS as potential indicator of prefrontal tonic DA levels might influence the optimal DA levels required for the different task conditions. Task-dependent phasic DA may add to different baseline DA levels, thereby affecting the balance between flexibility and stability. Thus, potentially increased phasic DA due to a higher task demand in the 400 ms relative to the 500 ms condition may have added to an already higher DA tone in individuals with a lower DA-CGS, further increasing stability thereby somewhat reducing the ability to flexibly update and respond extremely rapidly to task-relevant information in the 400ms condition. In the 500 ms condition these individuals may profit more from their higher baseline stability as a strong flexibility is comparatively less required than a stronger stability. However, in those with a relatively lower DA tone (higher DA-CGS) showing higher baseline flexibility such phasic DA responses may have driven the balance between flexibility/stability slightly towards stability. Due to their lower DA tone this may have led them toward a better balance between flexibility and stability in the 400 ms while in the 500 ms condition this balance is possibly less close to the optimum.

### ERPs and the DA-CGS

Consistent with previous research, we found the NoGo-N2 and NoGo-P3 to be enlarged in No-Go compared to Go trials. The NoGo-N2 also showed the expected fronto-central distribution and the NoGo-P3 a typical shift towards more anterior electrodes (Bokura et al., 2001; Bruin, Wijers, & van Staveren, 2001; Donkers & van Boxtel, 2004; Falkenstein et al., 1999; Jodo & Kayama, 1992).

In terms of the DA-CGS, similar to the behavioral results, there was a significant interaction between task condition and DA-CGS on NoGo-P3, supporting that P3 amplitude is partly DA modulated (Gallinat et al., 2003; Pogarell et al., 2011). Individuals with a higher DA-CGS (lower tonic DA tone) showed a smaller NoGo-P3 in the 500 ms condition than in the more demanding/rapid 400 ms one, whereas for those with a lower DA-CGS (higher DA tone) the opposite pattern was observed. Along with the interpretation of the behavioral data, our findings may suggest that DA-CGS-dependent variation of DA in frontostriatal circuits of cognitive control might have modulated the balance between cognitive stability and flexibility (Bilder et al., 2004; Cools & D'Esposito, 2011; Grace, 1995). That is, a DA-CGS-related lower or higher DA tone in the PFC would either promote cognitive flexibility or stability, depending on the specific requirements of the 400 ms and 500 ms Go/No-Go conditions, as detailed above. These DA-CGS-dependent variations in performance could then be accordingly reflected in NoGo-P3 amplitude. In line with this, larger relative to lower NoGo-P3 deflections have been related to more effective inhibition performance, and vice versa (Enriquez-Geppert et al., 2010; Falkenstein et al., 1999; Fisher et al., 2011). However, as discussed above, a task demand-dependent DA release (Aalto et al., 2005; Westbrook & Braver, 2016) may have added to the DA-CGS-related tonic/basal DA levels, thereby additionally influencing the balance between flexibility and stability (Cools & D'Esposito, 2011), in turn affecting task performance and NoGo-P3 amplitude.

No association between the DA-CGS and the NoGo-N2 was observed. This may support the notion that the NoGo-P3 could be more directly linked to motor response inhibition than the NoGo-N2 (Enriquez-Geppert et al., 2010; Janette L. Smith et al., 2007). However, the lack of association of the DA-CGS and the NoGo-N2 does not necessarily mean that this ERP is not DA modulated, rather, the investigated polymorphisms combined in the DA-CGS may have a greater impact on brain structures involved in generating the NoGo-P3 that diverge from those of the NoGo-N2 (Beste, Willemssen, Saft, & Falkenstein, 2010; Huster, Enriquez-Geppert, Lavallee, Falkenstein, & Herrmann, 2013).

### Limitations and future research

Genetic variations, even combined in a CGS, may yield comparatively small effects. However, a post hoc power analysis using G*Power (Faul, Erdfelder, Lang, & Buchner, 2007) showed that with our final sample size of N = 128 and an *α* of .05 we achieved a statistical power (1-*β* error probability) of ≥0.99. This decreases the likelihood that effects of the DA-CGS on the behavioral measures and particularly on NoGo-P3 amplitude, as intermediate phenotype (Gottesman & Gould, 2003) of response inhibition, have been overlooked.

Furthermore, our design does not allow directly disentangling to what extent task demand itself influences the DA response and may add to the baseline DA levels. Future research could investigate this issue for example by using a Go/No-Go task with more than two levels of task difficulty to better delineate a possible inverted u-shape function of DA action on performance. In addition, tasks tapping cognitive stability/flexibility could be used to examine relationships with the Go/No-Go conditions at hand. That is, the 400 ms Go/No-Go condition that putatively requires a higher flexibility should stronger be related with cognitive flexibility tasks (e.g., shifting/task-switching paradigms) than the 500 ms condition. Moreover, the used CGS approach, which potentially combines several distinct effects arising in different parts of the brain, does not directly allow to assess the results at this level of anatomical granularity, which limits such interpretations of the results. In addition, DA effects in frontostriatal pathways, as outlined above, are differentially modulated by D1 and D2 receptor function and expression (Frank, Seeberger, & O'Reilly, 2004). However, such effects were not examined in the present study.

Because our sample almost exclusively consisted of young students, effects may differ in more age-diverse samples, which could be examined in further studies. Female participants’ hormonal state might have also introduced some variability in our results, as DA levels are known to vary across the menstrual cycle (Becker, 2000). Moreover, it has to be noted that the interaction effect of DA-CGS × task condition on P3 amplitude would not hold the very conservative Bonferroni correction when tested two-tailed (*p* < 0.017; i.e., 0.05/3) as our main hypotheses were tested in three different models regarding %FA, RT, and the P3 amplitude, whereas the other analyses were presented only for descriptive reasons. However, the interaction effect of DA-CGS × task condition on P3 amplitude is consistent with those observed at the performance level (%FA and RT) and both findings correspond with explanations of DA function in cognitive control. Thus, the likelihood of a Type 1 error appears to be low.

## Conclusions

Overall, specific alleles, that are *DRD4* VNTR 7R, *DAT1* VNTR 10R, and *COMT* 158val, may contribute to impulsive behavior and a lower DA tone, as outlined above. However, it is often difficult to detect their effects due to small effect sizes of single polymorphisms and insufficient statistical power when investigating multiple genetic variants simultaneously. Our results suggest that using the DA-CGS leads to more consistent outcomes regarding response inhibition than the single polymorphism approach. Using a Go/No-Go task varying in difficulty, we could show that individuals with lower tonic DA activity (higher DA-CGS) rather profit from the more demanding rapid condition that might require a higher cognitive flexibility within the dynamic balance between stability and flexibility. In contrast, individuals with higher DA levels (lower DA-CGS) showed better performance and more pronounced NoGo-P3 amplitudes in the relatively easier condition that compared to the very demanding condition may rather benefit from cognitive stability. Additionally, a task demand-related DA release may have added to DA-CGS-dependent baseline/tonic DA level, possibly contributing to shifts in the balance between flexibility and stability. Overall, we think the cognitive stability/flexibility approach and the assumption of optimal DA levels depending on type of task or task condition could provide a plausible framework to tentatively explain the comparatively consistent behavioral and neurophysiological effects in our study. Moreover, the results suggest that depending on the characteristics of the situation the examined alleles could also pose an advantage and thus might better be seen as “plasticity alleles” (Belsky et al., 2009).

## Electronic supplementary material

ESM 1 (DOCX 2216 kb)
