# Supplementary material for: Cumulative Dopamine Genetic Score predicts behavioral and electrophysiological correlates of response inhibition via interactions with task demand
Source: Cogn Affect Behav Neurosci. 2019 Dec 4;20(1):59–75. doi: 10.3758/s13415-019-00752-w (PMC7012812; doi:10.3758/s13415-019-00752-w)
Supplement: Supplementary file 1 — (DOCX 2216 kb) [file 13415_2019_752_MOESM1_ESM.docx]

Supplementary material

Figure 2 of the manuscript represent the effect of DA-CGS on behavioral performance (%FA and RT) and NoGo-P3 as regression lines across the two conditions and thus are limited to the trend of the data. As two reviewers deemed it important to also depict the individual values beside the regression line, we calculated difference scores of the measures in the two conditions by subtracting %FA, RT, and NoGo-P3, respectively, in the less demanding 500ms condition from values in the 400ms condition. Thus, positive values represent better performance (lower RT, %FA, and NoGo-P3, respectively) in the 500ms than in the 400ms condition whereas the opposite is true for negative values in the difference score. The difference scores were then plotted against DA-CGS (see Figure). In line with Figure 2 of the manuscript, Figure 1 shows a trend of individuals with high DA-CGS towards less impulsive behavior in the 400ms compared to the 500ms condition as indicated by negative %FA and positive RT and NoGo-P3 difference scores. Contrary, individuals with lower DA-CGS show a trend towards less impulsive behavior in the 500ms than in the 400ms condition as indicated by positive %FA, and negative RT and NoGo-P3 difference scores.


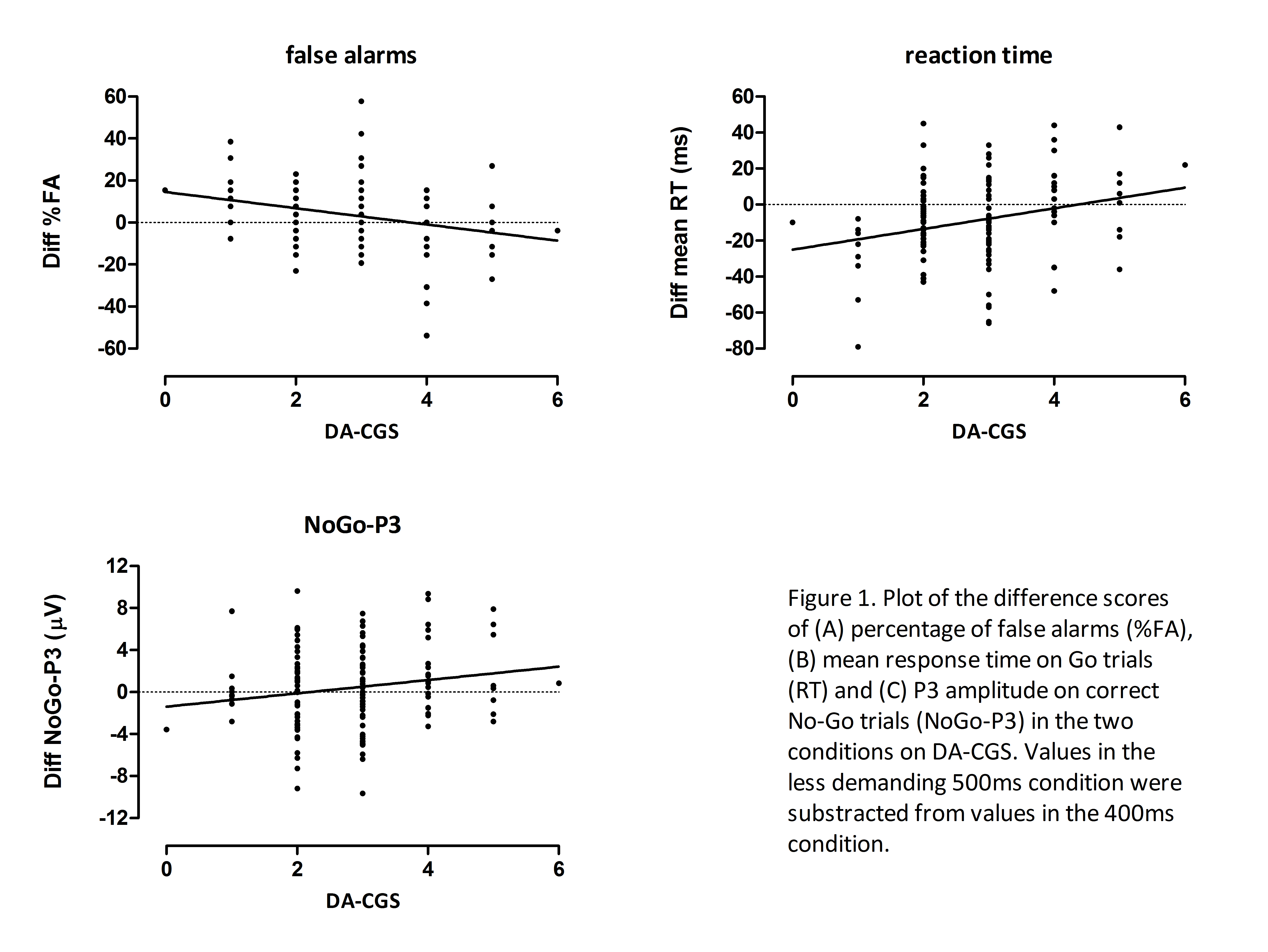


**A**

**B**

**C**
